# Lemierre’s syndrome complicating deep neck abscess: a case report

**DOI:** 10.1186/s12959-023-00543-x

**Published:** 2023-09-15

**Authors:** Yi Xu, Qingyuan Shi, Haiyue Ying

**Affiliations:** Department of Otolaryngology, Ningbo No.2 Hospital, Ningbo, 315000 Zhejiang China

**Keywords:** Lemierre’s syndrome, Streptococcus anginosus, Deep neck space infection

## Abstract

**Background:**

Lemierre’s Syndrome is a severe medical condition that can result from oropharyngeal infection, typically caused by Fusobacterium necrophorum, leading to sepsis, internal jugular vein thrombosis, and metastatic septic emboli. However, there is limited literature on this syndrome caused by Streptococcus anginosus, and few previous cases have been reported to have deep neck space infection. We present the first case of Lemierre’s Syndrome caused by Streptococcus anginosus with deep neck abscess.

**Case presentation:**

A 53-year-old male patient with no significant medical history presented with right neck pain after accidentally swallowing a fish bone one month ago. Laryngoscopy did not reveal any abnormalities. Five days prior to admission, the patient developed high fever. Imaging studies showed internal jugular vein thrombosis and a neck abscess surrounding the carotid artery sheath. Blood culture results were positive for Streptococcus anginosus infection, and the patient was diagnosed with Lemierre’s syndrome. The patient underwent surgical drainage and received antibiotics and anticoagulant therapy, and had satisfactory clinical progress. He was discharged after a 16-day hospitalization.

**Conclusions:**

Although Lemierre’s syndrome is rare, it needs attention because it can lead to serious complications and requires timely treatment. Deep neck space infections can be life-threatening and doctors must be aware of its potential severity.

## Background

Deep neck space infection (DNSI) refers to an infectious disease occurring in the fascial layers and spaces below the superficial cervical fascia, including cellulitis and abscess. It is commonly caused by infections of the mandibular teeth, tonsils, salivary glands, middle ear, sinuses, and upper respiratory tract [1]. The rate of complications and mortality can reach 40% when the infection spreads to the mediastinum, but with the widespread use of antibiotics, the mortality rate has significantly decreased [2]. Although most oropharyngeal infections are self-limited, they can still spread through fascial and deep neck spaces and eventually descend to the mediastinum. Due to the complex anatomy of the head and neck, DNSI can present with diverse clinical manifestations, making early diagnosis challenging and potentially leading to severe complications such as airway obstruction, Ludwig’s angina, necrotizing fasciitis, mediastinal abscess, sepsis, and jugular vein thrombophlebitis.

Lemierre’s syndrome(LS), also known as the “forgotten disease,“ is a rare disease [3] characterized by septicemia, internal jugular vein thrombosis, and metastatic septic emboli, which occur as a complication of oropharyngeal infections. It was first systematically described by the French microbiologist André Lemierre [4] in The Lancet in 1936. The most common pathogenic bacteria involved in this syndrome is Fusobacterium necrophorum [5].

The current study details a case of LS caused by Streptococcus anginosus, involving an atypical finding of an isolated deep neck abscess. This case is noteworthy due to the involvement of a less commonly pathogen and the presence of a rare complication. We aim to provide a detailed description and discussion of our clinical approach, in order to expand the existing data and knowledge about this specific disease.

## Case presentation

**Fig. 1:**
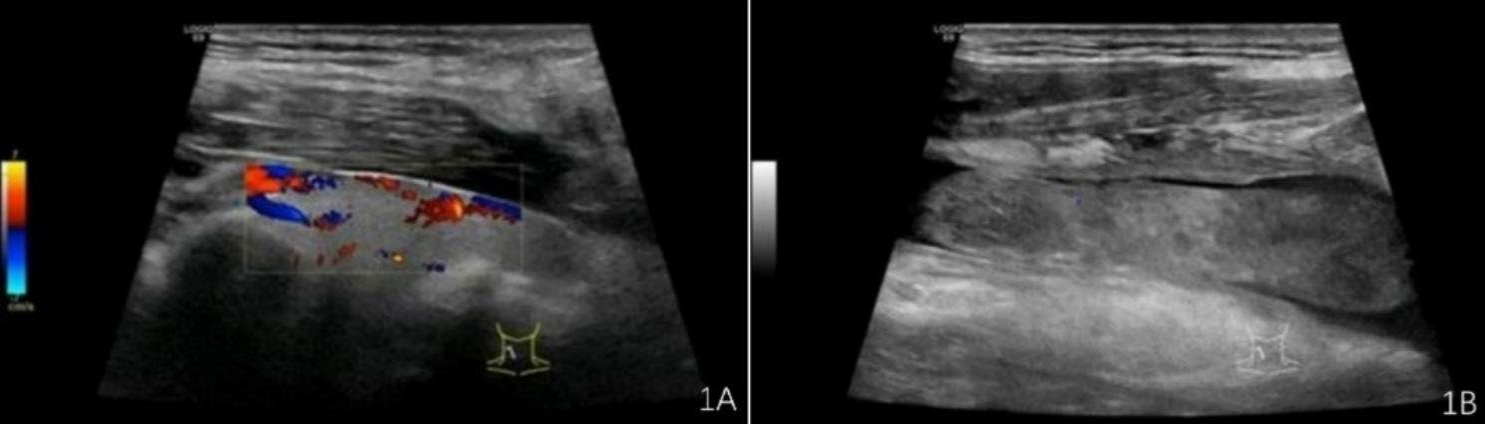
Strong echoes in the right side of the neck, suggesting the presence of a foreign body(**1A**), as well as the formation of a blood clot in the right internal jugular vein(**1B**)

**Fig. 2:**
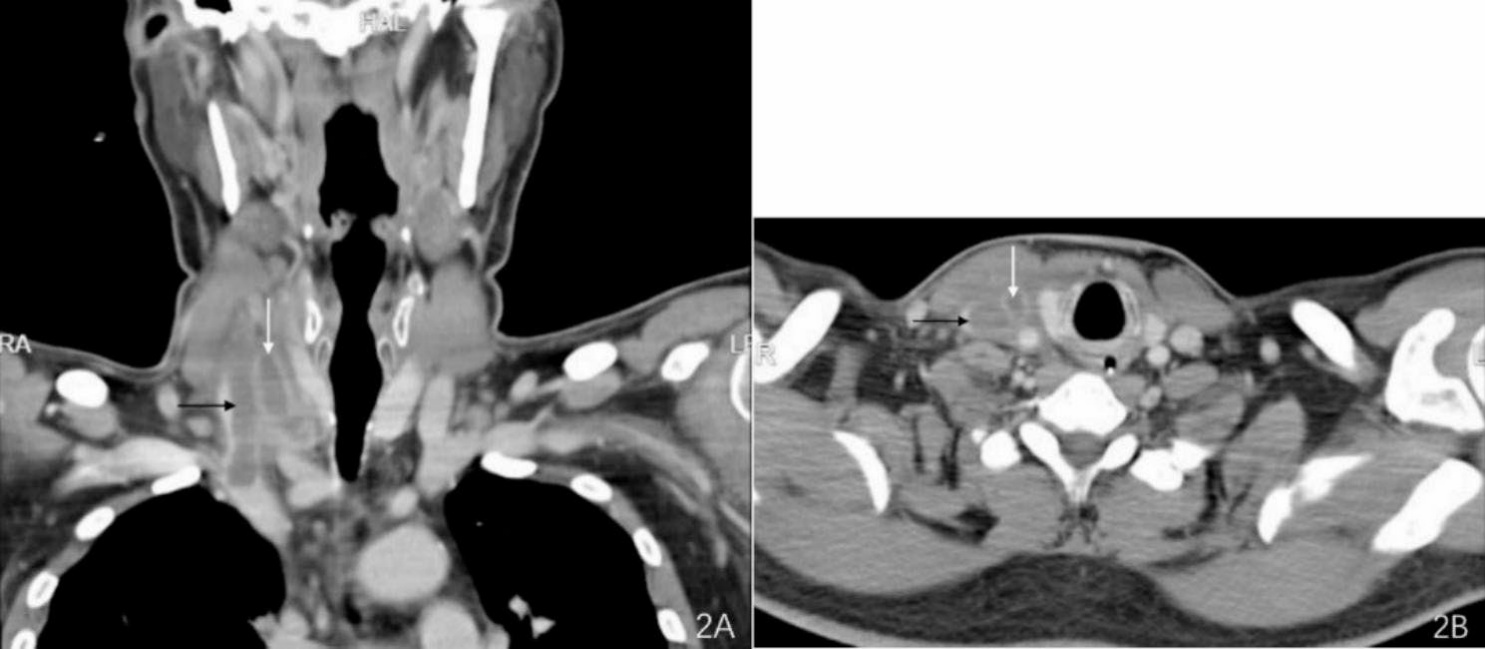
An intraluminal Peripheral filling defect in the right internal jugular vein, indicating the presence of a blood clot (see Black Arrow). A neck abscess can be seen surrounding the carotid sheath (see White Arrow)

**Fig. 3:**
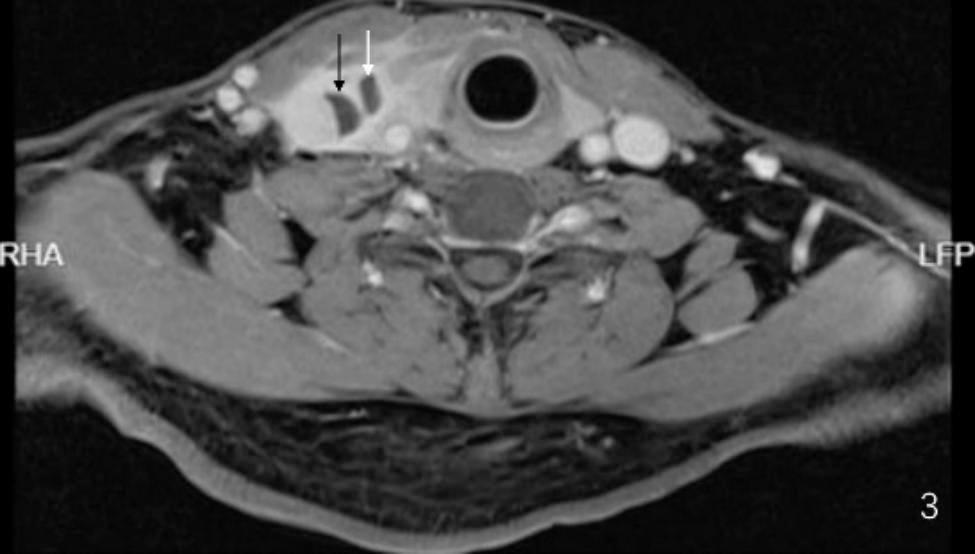
Abnormal signals around the carotid sheath in the right side of the neck, adjacent to the formation of a blood clot in the internal jugular vein. Multiple small-to-moderate lymph nodes are present on both sides of the neck

**Fig. 4:**
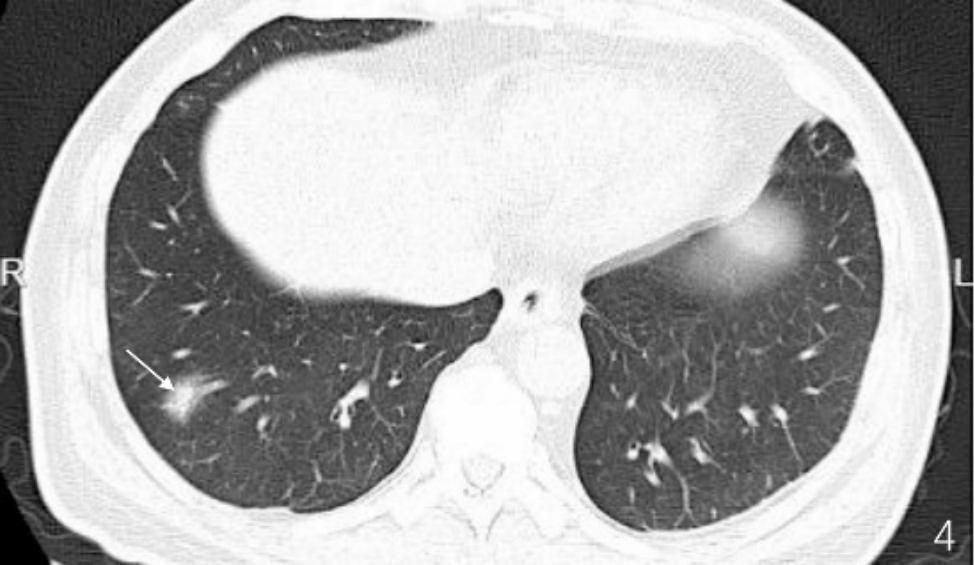
Mixed ground-glass opacities in the lower lobe of the right lung, possibly due to inflammation, with a small amount of fluid in both pleural cavities

**Fig. 5:**
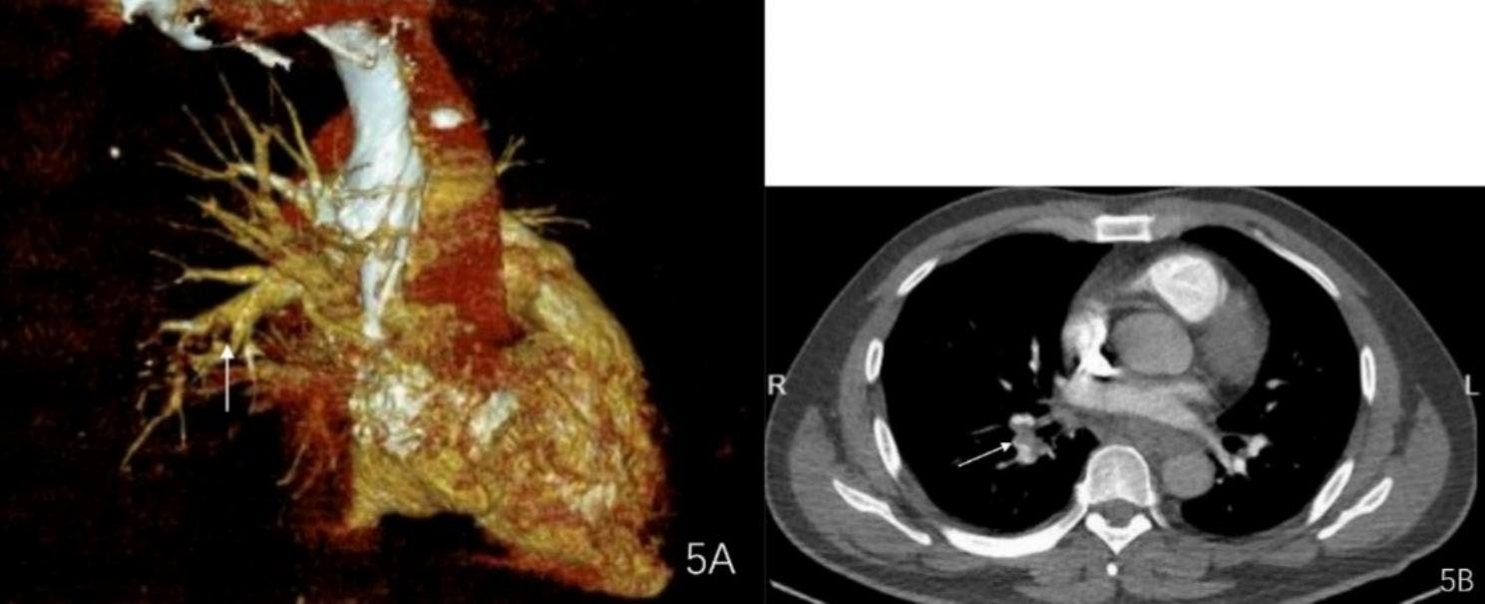
Multiple small blood clots in the distal branches of the pulmonary arteries in the lower lobes of both lungs

**Fig. 6:**
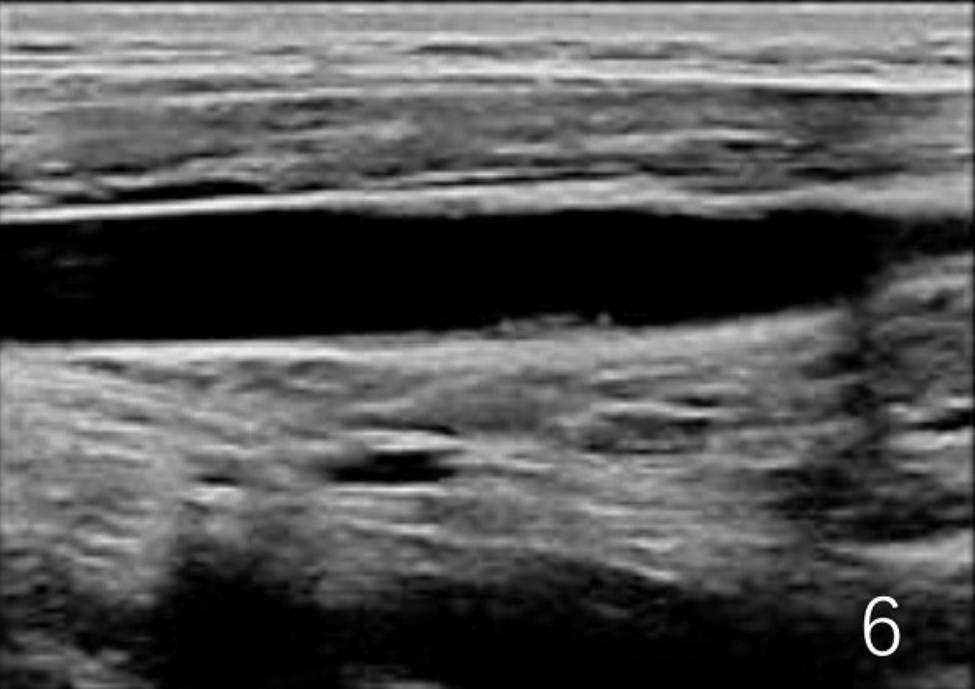
Compared to Fig. 1B, partial re-opening of the right internal jugular vein

A 53-year-old male patient without significant medical history or allergy history accidentally swallowed a fishbone in April 2022 and developed right neck pain. A local hospital performed a laryngoscopy which showed no abnormalities and the patient was not given any treatment, but was instructed to observe at home. One month later, the neck pain worsened and the patient developed a high fever, so he was admitted to our department for treatment. The neck ultrasound showed strong echo on the right side of the neck with an unclear foreign body (see Fig. 1A), and thrombosis in the right internal jugular vein (see Fig. 1B). Laboratory tests upon admission showed decreased platelet count (75*10^9^/L), increased white blood cell count (20.4*10^9^/L), elevated C-reactive protein (379.22 mg/L), elevated D-dimer (2573.0ng/ml), normal prothrombin time(12.2s)and normal activated partial thromboplastin time (34.6s). The patient had no history of intravenous drug use or recent travel nor any known exposure to sick patients. Blood culture obtained upon admission showed Streptococcus anginosus infection. Contrast-enhanced CT of the neck showed an endoluminal filling defect in the right internal jugular vein, suggesting the presence of thrombus, and an abscess surrounding the carotid artery sheath (see Fig. 2A and B). Contrast-enhanced MRI of the neck showed abnormal signal around the carotid artery sheath on the right side, adjacent to thrombosis in the right internal jugular vein, and multiple small-mildly enlarged lymph nodes on both sides of the neck (see Fig. 3). CT of the chest showed scattered ground-glass nodules in the lower lobe of the right lung, suggestive of inflammation, and a small amount of pleural effusion on both sides of the chest (see Fig. 4). Pulmonary artery CTA revealed multiple small thrombi formation in the distal branches of bilateral pulmonary arteries in the lower lobes (see Fig. 5A and B). The diagnosis was LS, which is characterized by thrombotic venous inflammation in the internal jugular vein and disseminated infection manifestation (usually septic pulmonary embolism), which usually occurs after recent oropharyngeal infection. The patient received intravenous therapy with piperacillin-tazobactam, vancomycin, and rivaroxaban for anticoagulation. Meanwhile, abscess drainage surgery was performed, but no foreign body was found and the thrombosis in the internal jugular vein was not treated. The patient’s fever and neck tenderness improved, and he recovered well. He stayed in the hospital for 16 days and was discharged on oral antibiotics and anticoagulation therapy. The duration of anticoagulation therapy was 6 months, and the patient had a follow-up ultrasound examination after 1 month of treatment, which showed no changes in thrombosis in the internal jugular vein. 9 months after surgery, the patient underwent a follow-up ultrasound (see Fig. 6) which showed partial recanalization of the right internal jugular vein.

## Discussion

Thrombophlebitis of the internal jugular vein is a rare but life-threatening complication of DNSI, also known as LS. The formation of blood clots is related to hemodynamic changes, increased blood viscosity, and endothelial damage [6]. The cause of thrombus formation in this patient may be related to direct compression of the internal jugular vein by the abscess, leading to hemodynamic changes. In addition, it may also be related to endothelial damage. Following endothelial damage, subendothelial collagen becomes exposed, facilitating platelet adhesion to the collagen surface. This process triggers platelet signaling pathways, resulting in platelet activation and the release of endogenous ADP and TXA2. Consequently, additional platelets are recruited to adhere to one another, leading to irreversible platelet aggregation. Concurrently, the coagulation system is activated, inducing localized blood coagulation. Soluble fibrinogen in the plasma is converted into insoluble fibrinogen, ultimately forming a blood clot to halt bleeding. The patient had a history of a fishbone injury in the pharynx, suggesting a pharyngeal source of infection. The possible pathogenesis is that bacteria or viruses damage the mucosal barrier of the oropharynx, enter the cervical space, and then spread to the internal jugular vein through direct extension, lymphatic and hematogenous dissemination, leading to phlebitis. The endothelium of the vein is damaged, the pathogen activates platelets, activates the coagulation cascade, and thus forms a thrombus [7]. The lungs are the most common site of metastasis, accounting for 85% of all secondary infections. Common pulmonary lesions are necrotic cavities, but they can also present as pneumonia, pleural effusion, empyema, lung abscess, and necrotizing mediastinitis [8, 9]. This patient presented with pulmonary embolism, pneumonia, and a small amount of pleural effusion in the lungs. Due to timely antimicrobial and anticoagulant therapy, the patient’s condition did not worsen, avoiding possible fatal diseases. Due to the destructive effect of hemolysin produced by the pathogenic bacteria, the consumption of thrombus formation, and bone marrow suppression, the patient’s platelet count often decreases and clotting disorders may occur [10]. The most common pathogen of LS is Fusobacterium necrophorum. Although the positive rate of blood culture for F. necrophorum is low, the detection of F. necrophorum and other pathogenic bacteria in blood culture is still an important diagnostic criterion for LS. Therefore, blood culture should be performed early for febrile patients to identify pathogens. The blood culture of this patient was positive for streptococcus anginosus. To our knowledge, there are few reports of cases of streptococcus anginosus infection, and there are also few reports of deep cervical abscesses occurring in cases like our patient. The early symptoms of LS lack specificity. If there is persistent high fever or neck tenderness, LS should be highly suspected [11]. B-ultrasound is convenient, radiation-free, and can quickly detect the location and size of cervical venous thrombosis, which is of great significance for diagnosis [12], but its sensitivity for deep cervical tissue and newly formed thrombus is low [13]. Contrast-enhanced CT is the best method for diagnosing LS because it can not only show internal jugular vein thrombosis but also reveal complications such as pulmonary embolism, empyema, osteomyelitis, and brain abscesses, and epidural abscesses [14]. It is often used as a gold standard to evaluate the scope of soft tissue infections in the neck [2]. In addition, contrast-enhanced MRI has good soft tissue contrast and multi-planar relationships, which can well distinguish the relationship between blood vessels, abscesses, and adjacent soft tissues.

The treatment of LS mainly includes antimicrobial therapy, surgical intervention, and anticoagulation therapy. Antibiotics that are resistant to β-lactamase hydrolysis and have anaerobic activity should be selected [11, 14], and the treatment course is 3–6 weeks [8]. Surgical intervention can be used for severe cases, which can prevent the production of further septic emboli and should be performed by the corresponding surgeon who is in contact with the abscess site, including periodontal disease treatment and abscess incision and drainage. If the abscess is not controlled, it may develop into a severe cervical and facial soft tissue infection [15]. Regarding abscess treatment, in this patient, immediate empiric treatment with intravenous broad-spectrum antibiotics covering anaerobic bacteria were prioritized until the pathogenic bacteria were identified. As inflammatory markers significantly decreased after antimicrobial therapy, the antibiotics were not changed. In addition, surgical drainage was performed on the infection site. Due to the low incidence of LS, it is difficult to obtain randomized controlled studies to verify the risks and benefits of anticoagulation therapy. Anticoagulation therapy is still controversial, but it is recommended to use anticoagulation therapy for expanding thrombus scope, persistent high fever, or the appearance of retrograde cerebral venous sinus thrombosis. The treatment course is 6–12 weeks [16]. The American College of Chest Physicians’ 2012 guidelines recommend anticoagulation therapy for 3 months in patients with non-progressive cervical internal venous thrombosis, which can reduce the risk of recurrent thrombotic embolism [17]. For patients who cannot control the condition even with adequate antibiotic and anticoagulation therapy, ligation or surgical removal of the internal jugular vein can be selected. In this case, the patient’s thrombus did not expand after antimicrobial and rivaroxaban anticoagulation therapy. The internal jugular vein thrombosis measures approximately 4.5 cm in length, extending from the upper segment below the bifurcation of the common carotid artery to the lower segment below the subclavian vein. Due to the significant surgical complexity and the associated high risk, ligation of the thrombosed vein was not chosen as the preferred approach.

## Conclusions

If a DNSI patient presents with persistent high fever or neck swelling and conservative treatment is ineffective, LS should be highly suspected. Enhanced CT or ultrasound can provide a definitive diagnosis. Early diagnosis and treatment are the key to a good prognosis. In our case, the patient recovered well with antibiotic and anticoagulation therapy as well as surgical drainage of the abscess, avoiding severe complications. LS should not be overlooked, as it is a treatable condition.

## Data Availability

Supporting data is available.

## List of abbreviations

DNSI: Deep neck space infection
LS: Lemierre’s syndrome
